# 0996. Improventment of method primary cultivation and identification of rat pulmonary microvascular endothelial cells

**DOI:** 10.1186/2197-425X-2-S1-P81

**Published:** 2014-09-26

**Authors:** L Yong-Jun, M Jie, W Ping-Ping, O Bin, C Juan, G Xiang-Dong

**Affiliations:** Department of SICU, The First Affiliated Hospital, Sun Yat-sen University, Guangzhou, China; The First Affiliated Hospital, Sun Yat-sen University, Guangzhou, China

## Introduction

Pulmonary microvascular endothelial cell(PMVEC) is a very important tool to study acute respiratory distress syndrome. Several methods for *in vitro* cultivation of PMVEC have been established, but all the methods damage PMVECs either by use of proteolytic emzymes or by mechanical trauma. This study was to improve the method for primary cultivation of rat pulmonary microvascular endothelial cells and identify the primary cultivated cells.

## Objectives

To improve the method for primary cultivation of rat pulmonary microvascular endothelial cells and identify the primary cultivated cells

## Methods

Pulmonary microvascular endothelial cells were derived from peripheral lung tissue of Sprague-Dawley rats. Primary cultivation method was improved from the procedures of animal selection, lung tissue perfusion, tissue piece pasting, culture medium selection and so on. Inverted microscope was used to observe morphological characteristics of pulmonary microvascular endothelial cells. Immunohistochemical staining for expression of VIII-related antigen and CD31, and observed by fluorescence microscope for binding with lectin from BSI (FITC-BSI binding away ) to identified pulmonary microvascular endothelial cells. The cell purity was detected with flow cytometry.

## Results

The PMVECs were exhibited as polygon and presented typical cobblestone-like morphology after fusion to monolayer with contact inhibition. PMVECs turned to be fusiform and presented a swirling or aggregate growth pattern after transfer of culture. Immunohistochemical staining revealed that the expression of CD31 and factor VIII-related antigen was positive. Besides, there were positive findings for FITC-BSI assay. The vitality and growth rate of PMVECs were in good condition. The cell purity was 93.2% with the improvement method of primary cultivation.

## Conclusions

The improved method for primary cultivation is easy to handle, with favorable repeatability and success rate. PMVECs obtained with this method will display lower contamination, higher purity, faster growth and better status.
